# Telerehabilitation in the Finnish Outpatient Rehabilitation Setting from the Perspective of the Socio-Technical Systems Theory

**DOI:** 10.3390/ijerph20156519

**Published:** 2023-08-03

**Authors:** Tuija Partanen, Riitta Seppänen-Järvelä, Sinikka Hiekkala, Jari Lindh

**Affiliations:** 1 Kela Research, Social Insurance Institution of Finland, FI-00250 Helsinki, Finland; riitta.seppanen-jarvela@kela.fi; 2 Faculty of Social Sciences, University of Lapland, FI-96101 Rovaniemi, Finland; jari.lindh@ulapland.fi; 3 The Finnish Association of People with Physical Disabilities, FI-00280 Helsinki, Finland; sinikka.hiekkala@invalidiliitto.fi

**Keywords:** telerehabilitation, telepractice, COVID-19 pandemic, rehabilitation professionals, physiotherapy, occupational therapy, speech and language therapy, neuropsychology, music therapy, socio-technical system theory

## Abstract

Background: In the development of effective telerehabilitation (TR) interventions, understanding the various characteristics affecting its practice is essential. Remote connection creates a new technically shaped environment for therapy and, therefore, previous therapy methods do not work the same way as before. Objective: The objective of this survey was to describe the practice of TR through the socio-technical theory approach. Methods: The 629 respondents to the online questionnaire included music therapists, occupational therapists, speech and language therapists, physiotherapists, and neuropsychologists. The materials consisted of five open-ended questions. The analysis combined data-based and theory-based analysis. Results: In the data-based content analysis, we identified three main categories and eight generic categories, whereas in the theory-based, we categorised the main results according to the Fit Between Individuals, Tasks, Technology, and Environment (FITTE) framework dimensions. TR is everyday-life based, it requires shared participation, and the approach has to include coaching and collaboration with the client and their close associates. The everyday-life environment is one of the main dimensions that affect all the other dimensions. Conclusions: TR can be seen as technology-mediated home-based rehabilitation, as it can integrate rehabilitation into the client’s everyday life. In TR, therapy becomes multilateral and it creates a new kind of shared partnership into outpatient therapy.

## 1. Introduction

COVID-19 accelerated the adoption of telehealth solutions in many healthcare services [1]. Telerehabilitation (TR) is defined as the delivery of rehabilitation intervention using a variety of telecommunication technologies (including mobile phones and video calls) without face-to-face interaction between a therapist and the client. TR can be synchronous, asynchronous, or it can combine both ways as a hybrid model [2,3,4]. Before the pandemic, TR was provided quite rarely [1,5]. However, once the pandemic started, professionals had to rapidly shift their services to TR [6,7]. Some could say that the COVID-19 pandemic caused a major change in how therapists practice [7]. For example, over half of the music therapists, neuropsychologists, physiotherapists, occupational therapists, and speech and language therapists who participated in the Finnish survey took up TR with all or most of their clients at the beginning of the COVID-19 pandemic [3]. A year later, 48% of physiotherapists in the USA were providing TR sessions at least occasionally [5].

In recent reviews, practical barriers (e.g., lack of physical guidance) and technical barriers are perceived as major challenges to the successful adoption of telehealth services [1,4,8]. Despite the challenges, TR has proven to be an effective tool to complement traditional healthcare services and its most common advantages for clients are increased access and reduced traveling to rehabilitation services [1,4,8,9]. TR has the potential to improve home-based rehabilitation services [10,11] and support the clients’ participation in daily life [12] and for improving treatment outcomes [4]. Rehabilitation services are increasingly moving away from face-to-face delivery models. Therefore, developing effective remotely delivered interventions and understanding different factors affecting their optimal uptake is crucial [13,14]. The shift to remote-based rehabilitation services involves changes far greater than replacing the mode of delivery [15]. 

Recent studies have described factors affecting the optimal practice of TR in rehabilitation settings [13,14,15]. An ongoing challenge of putting TR technologies into practice is to operationalise their use within the complex rehabilitation system [16]. Studies of TR usually focus on intervention outcomes and narrowly pay attention to the multifaceted socio-technical elements of TR [15]. Earlier studies on the use of TR in rehabilitation have mainly focused on interventions, acceptability, feasibility, and attitudes towards TR [4,8,13,17,18]. Remote connection creates a new technically shaped environment for therapy and, therefore, previous therapy modes cannot be used the same way as before. Research is needed on the relationships between human and social factors and TR technology.

Taking up technological solutions produces predicted and unpredicted changes in the rehabilitation practice. Even though the importance of this change is recognised in the practice context, various critical factors can still ease or complicate the uptake of relevant technologies [16]. This transition towards TR in rehabilitation services can be seen as a systemic challenge [15,16]. Therefore, the complex socio-technical effects of TR have to be studied in different rehabilitation settings. 

The socio-technical approach [19,20] provides a diverse framework for the study of TR. It considers the characteristics of the clients and therapists, relevant technologies, tasks in therapy practice, and the environment in which TR is carried out. The socio-technical approach broadens the perspective on TR, enabling the identification of its strengths, shortcomings, and areas for improvement. Without this kind of research reflecting on the reasons for the success and failure of TR technology, attention can be drawn to irrelevant matters.

This study aims to describe the main dimensions and practice of TR through means of the socio-technical theory and answer the following questions: How is telerehabilitation put into practice from a socio-technical perspective? Which dimensions are highlighted in the practice of telerehabilitation? In this study, the practice of TR in outpatient rehabilitation at the beginning of the COVID-19 pandemic was examined from the sociotechnical perspective [19,20]. This study produces new knowledge on the complex context of TR by adopting a socio-technical approach, especially the Fit Between Individuals, Tasks, Technology, and Environment (FITTE) framework. The novelty of this study is in combining social and technical systems, and its results reveal the essential principles in conducting individual outpatient therapies through remote connection.

## 2. Materials and Methods

### 2.1. Study Approach

This study explores TR from the point of socio-technical systems theory using the FITTE framework [20]. The FITTE framework is based on the FITT framework [19], which encompasses the interactions between users and technology, tasks and technology, and users and tasks. The framework explores the human factors in technical solutions. It is based on the idea that the adoption of technology in healthcare settings depends on the reconciliation of individual characteristics (e.g., knowledge of the technology, motivation), technology characteristics (usability and functionality), and tasks. According to the framework, a change in one part of the system will also result in changes in the other parts. The system as a whole can be understood from its interactions between the various parts of the system [19,20]. 

Environmental and contextual factors have been noted to affect the use of technology and, therefore, researchers proposed to extend the FITT framework to include an environmental dimension [20,21]. Environmental attributes include settings (e.g., timing of activities), physical environments (space, layout, materials), and organisational policies and procedures. The environmental dimension may help to explain why a certain technology works in one setting but not necessarily in another [20]. In this study, environments refer to the external factors in which a person’s everyday life occurs. These include the built environment and technology, natural environment, social and economic systems, and cultural environment [22,23].

In this study, everyday life refers to a complex consideration of different activities related to work, education, unpaid work, free time, and personal care [23]. Everyday life consists of daily habits and routines in relation to time and place [24]. The technical system of this study is interpreted as the context of TR. The technical system includes remote connections (e.g., internet or telephone connection), infrastructure, hardware (e.g., microphone, camera, image and sound quality, settings of the needed technical instruments), and software (e.g., videoconferencing, applications).

The social system is interpreted as the context of the outpatient rehabilitation services in Finland and the client–therapist interaction. According to O’Keeffe et al. and Babatunde et al. [25,26], the main characteristics of the client–therapist interaction are communication and interaction, partnership, the therapists’ practical skills, and client-centred rehabilitation and its goals. In addition, organisational and environmental factors, expectations and therapy outcome, and the therapists’ and clients’ roles and responsibilities greatly affect this interaction. A client’s personal characteristics, such as life experiences, willingness to engage, and existing resources are also relevant [25,26]. 

### 2.2. Study Setting and Participants

This study was conducted in Finland using an online survey. The current study is part of an ongoing research project exploring the quality of individual therapies and the change in the relationships between clients and therapists. Ethical approval for the main study (following the tenets of the World Medical Association Declaration of Helsinki) was obtained from the Ethical Review Board of the Social Insurance Institution of Finland (Kela) in Helsinki (protocol code 4/1/500/2019 22 November 2019). 

The focus of this survey was on outpatient therapy with intensive medical rehabilitation clients in Finland. Kela provides access to different types of therapies for intensive medical rehabilitation clients. Eligible clients must have a diagnosed illness or impairment that significantly limits their functioning and complicates their daily lives [27]. The context of this study was the first wave of the COVID-19 pandemic, a time when professionals were taking up TR with quite limited experience with it. The first part of the study investigates and compares the uptake of TR in Finland amongst different rehabilitation professions during the COVID-19 pandemic [3]. 

The target population comprised professionals licensed to practice psychotherapy, physiotherapy, occupational therapy, speech and language therapy, music therapy, or rehabilitation neuropsychology who were providing outpatient therapy services either in the public or private sector. In the first part of the study, psychotherapists reported less challenges in the adoption of TR than the other professionals reported [3]. Compared to psychotherapy, other outpatient therapies are more activity-based, and, therefore, the current study focuses on the responses from music therapists, neuropsychologists, physiotherapists, occupational therapists, and speech and language therapists. This study investigates different professional perspectives to provide insight on the extent to which there is consistency in the experiences of different professionals in adopting TR to their therapy practice. In total, 629 professionals answered the questionnaire (Table 1. Participants of the study). Of the respondents, 6% reported being male, 93% female, 0.5% other, and 0.5% did not want to clarify their gender. Furthermore, the respondents were divided in age groups as follows: 20–29 years old (6%), 30–39 years old (20%), 40–49 years old (29%), 50–59 years old (30%), and 60 or older (16%).

### 2.3. Data Collection

The online questionnaire was specially created for the COVID-19 pandemic, and it is illustrated in more detail elsewhere [3]. The survey was carried out from 7 May to 1 June 2020. The questionnaire did not contain any identifying information, and the respondents answered it anonymously. All questions in the questionnaire were voluntary and it was possible to skip any of them. The online questionnaire was distributed through email (to Kela’s service providers) and social media. In addition, the questionnaire was sent to various email groups through professional organisations. The current study’s materials consisted of five open-ended questions (Table 2. Material of the study). Open-ended questions allowed the respondents to elaborate or expand their aspects of TR or respond to a question in their own words [28]. We used a qualitative design with open-ended questions to elicit the respondents’ views and additional insights about the uptake and the practice of TR. Responses varied from single words to multi-sentence answers.

### 2.4. Data Analysis

**Figure 1:**
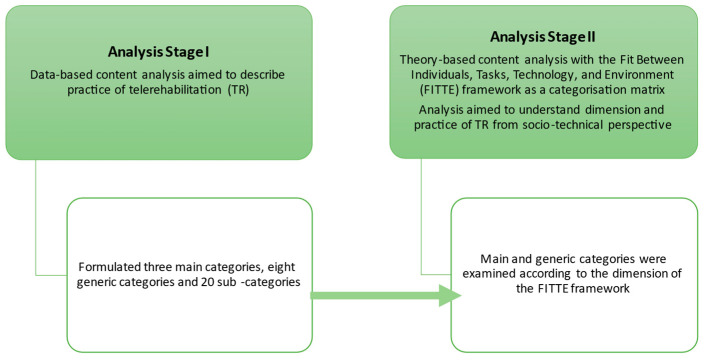
Stepwise progression of data analysis.

We combined data-based content analysis and theory-based content analysis (see Figure 1) [29]. In the first stage, we analysed the data with a data-based content analysis according to the suggested process [30,31]. In the content analysis preparation phase, the first author (TP) read all the texts in order to obtain an overview of the content. In the data, we found 657 quotations related to the aim of the research. Unit of analysis was a response and/or phrases containing aspects related to the research questions. TP condensed and formulated the unit of analysis into codes. In the organising phase, similar or related codes were grouped into 20 subcategories. Codes and subcategories were discussed with other authors. We grouped the subcategories into eight generic categories, which we thereafter abstracted into three main categories.

In the second stage, we used theory-based content analysis [30,31]. In order to describe how TR is put into practice from a socio-technical perspective, we used the FITTE framework [20] as a categorisation matrix. With the deductive analysis, we placed the main and generic categories that were formulated in the first stage of the analysis into the socio-technical theory context. We organised the main and generic categories into the FITTE framework dimension of environment and fits between task and technology, individual and task, and individual and technology. The conclusions formed as a result of the first stage of the analysis were conceptualised in relation to the FITTE framework. 

We conducted qualitative analyses with ATLAS.ti 22 software [32]. When used in a qualitative research process, digital software supports analytic activities and creates a systematic and organised approach to work and documentation [33,34]. In analysis, we used different ATLAS.ti analysis and visualisations tools to organise data and identify relationships from content of open-ended answers. TP conducted the qualitative analysis and other authors followed up on the analysis process and discussed the content of the categories and their interpretation.

## 3. Results

### 3.1. Data-Based Content Analysis

**Figure 2:**
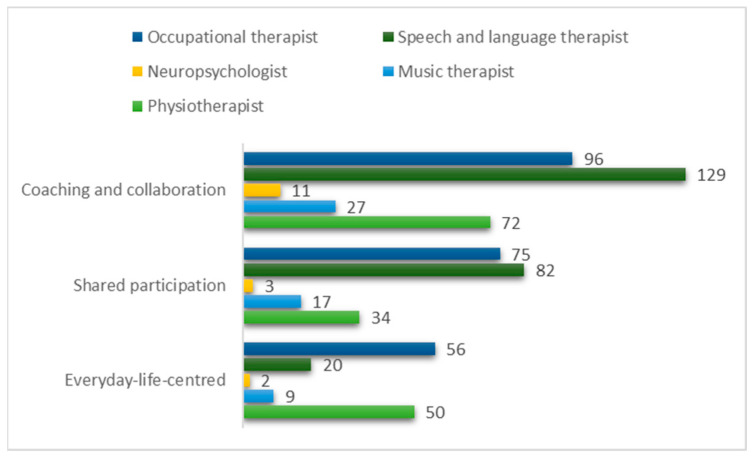
Quotations in main categories by professional groups.

In the data, respondents described the time during COVID-19 when they rapidly went from face-to-face rehabilitation to TR. The aim was to form a diverse description of therapists’ experiences in adoption of TR. The qualitative data analysis produced three main categories: everyday-life centred, shared participation, and coaching and collaboration, which provide insight into the respondents’ perspectives on the practice of telerehabilitation (see Figure 2).

Every main category has quotations from all professional groups. The most quotations were identified from the responses of speech and language therapists and occupational therapists. Neuropsychologist and music therapists’ responses were fewest, but their point of view has also been brought up in this study. The main categories consist of eight generic categories and twenty subcategories (see Table 3).

The first main category, Everyday-life centred brought up the meaning of everyday life and the environment where TR was practiced. TR took place in the client’s everyday life. As one of the respondents put it:

*Moving to telerehabilitation may have brought some new dimensions and aspects of everyday life to rehabilitation, which we were able to advance through the already developed process*.

*(Music therapist)*

Therapists felt that training remotely can be part of the client’s normal everyday life and everyday events. With the whole family at home, the therapist has been given a unique opportunity to peek into the client’s everyday life. Some respondents expressed that incorporating therapy into everyday life could still be strengthened, for example, through the therapist’s education. The respondents found that seeing clients train at home enabled them to provide information, useful hints, and instructions to clients concerning their everyday lives, as the following quotation elucidates:

*With some, our work approach included more guiding of everyday life. A lot of knowledge and information and tips were forwarded to everyday life*.

*(Music therapist)*

Some variation was found in the responses related to goals. Upon adopting TR, some of the respondents changed the goals of therapy, some broke the goals down into smaller parts, and some kept the goals similar to face-to-face therapy. Some of the respondents had changed the goals to be more concrete and related to home conditions. One respondent felt that goal-oriented working is not much different to face-to-face therapy, but practicing at home allows the goals to be tied more strongly to the client’s everyday life. Responses brought up that when goals are based on everyday life, training and exercises became a natural part of the client’s ordinary day. As one of the respondents explained:

*For the most part, the goals are everyday-life-oriented, which makes it possible to apply the methods in telerehabilitation too*.

*(Occupational therapist)*

TR took place in the clients’ everyday-life environments, mostly at their homes. Everyday environment may be a challenge to therapy intervention. Some respondents considered clinic-based practice to be the most suitable environment for therapy, especially when therapy was mainly activity-based or mobility training. Some responses brought up that facilities and therapeutic equipment are significant in activity-based intervention.

Safety issues should be taken into consideration when training takes place in the client’s everyday environment with remote guidance. One respondent elucidated:

*The more challenging exercises are not safe to perform remotely, and the equipment available at home is not versatile enough*.

*(Physiotherapist)*

The responses pointed out that safe training in everyday environments is not always possible without adjusting the activities and training. For example, therapists facilitated mobility exercises or training focused more on upper body rehabilitation. 

The second main category, Shared participation illustrated the meaning of a supportive person, human interaction and engagement, and inclusion of clients and their close associates. Many intensive medical rehabilitation clients need a supportive person to participate in TR. The need for assistance and support varied from minor assistance with opening the connection to continuous assistance during TR sessions. For example, one of the respondents said:

*With young rehabilitees, the supportive person and the parent play a big role in guiding the rehabilitee. If a supportive person could not be found in the rehabilitee’s everyday life for the duration of therapy, telerehabilitation could not be started*.

*(Music therapist)*

The supportive person’s competence to guide and assist during TR was important in order to enhance the client’s possibilities to participate.

The human interaction involved a third party (a supportive person), not usually present in a face-to-face therapy relationship. Some respondents faced challenges in the interaction between the client and their close associates and found it demanding to guide these situations through a remote connection. Respondents found that maintaining interaction with familiar clients was successful.

Engagement and inclusion of clients and their close associates is important in TR. The need for a supportive person in TR becomes an opportunity to involve the clients’ close associates in therapy situations. Respondents pointed out that the inclusion of clients’ close associates was possible in a more diverse way than before.

In adopting TR, more responsibility was shifted to the clients and their close associates. However, not all had the opportunity or resources to take on responsibilities such as opening the connection or layout and preparation of equipment needed during the therapy session. TR requires active participation from both the therapist and the client, as one of the respondents stated:

*With telerehabilitation, I’ve started to pay more attention to the parent as an active participant in therapy. Since I tend to engage the parent in teletherapy even without noticing, it is easier for them to take responsibility for the weekly exercises outside the sessions as well*.

*(Speech and language therapist)*

The third main category, Coaching and collaboration illustrated established practices as well as limitations in the use of methods and how therapists experienced communication within the frame of technology. In some cases, the client’s own training with a remote connection was not successful and, therefore, focus of the therapy shifted towards guidance. The guidance and coaching methods worked well, and the clients’ close associates had the opportunity to guide exercises. One respondent said:

*In day care centres, therapy can be implemented by personal assistants guiding the exercises. Works well with guided therapy, the child’s close associate can instruct*.

*(Occupational therapist)*

Modelling exercises for the clients’ close associates and committing them to therapy proved a natural part of TR. Guidance of professionals, families, and other close associates alongside therapy was perceived as more successful than expected.

TR was perceived to be well suited for self-directed training. Individualised instructions for self-directed training and home exercises were added to the client’s everyday life. Between the real-time therapy appointments (video/phone connection), the therapists supported self-directed training by text messages, pictures, and videos.

The respondents felt that the technological frame shaped the therapy practice. From behind the screen, some exercises were easier to perform than others, and the therapists had to carefully select the exercises suitable for the client’s situation. The respondents pointed out that sometimes clients did not have suitable technological equipment or equipment placement was not successful for therapy. Therefore, it was difficult to see the client’s whole body or movements. 

In TR, there are some limitations in the use of methods. The respondents found that observation, assessments, and evaluation were challenging to put into practice in TR. They also felt that these challenges arose because there were no previous ways to carry out the evaluation remotely. Manual guidance and tactile methods were the main limitations encountered during TR. For example, some of the respondents felt that pain treatment, ensuring the quality of exercises or performance techniques, and co-playing with the child on the floor were challenging to put into practice in TR. A respondent reported this limitation by stating:

*Other methods (e.g., homework) have been considered in the rehabilitation of visual-perceptual difficulties as demonstrating the exercise in practice during rehabilitation sessions is not possible in the same way during remote sessions*.

*(Neuropsychologist)*

Some respondents pointed out that in face-to-face meetings, the clients’ spontaneity and active participation with the whole body and all senses is more successful. Therefore, the respondents felt that TR alone cannot replace physical presence and shared activity. Although the importance of face-to-face contact was emphasised in the responses, TR was considered a good addition or an alternative method for therapy practice.

The respondents felt that the communication style within the frame of technology differs from face-to-face communication. Contact is framed by technology. As one respondent described:

*Body language does not come to its own in remote contact, and clients whose expression or speech comprehension needs to be interpreted and supported in many ways are not, in my opinion, suitable for telerehabilitation*.

*(Speech and language therapist)*

The importance of speech and language skills was strengthened due to limited possibilities to use gestures and nonverbal communication. The respondents pointed out that therapists should have a shared language with the clients and their close associates. Communication focused on spoken language and therefore a language barrier was perceived as more harmful than in face-to-face appointments.

### 3.2. Theory-Based Analysis with the FITTE Framework

**Figure 3:**
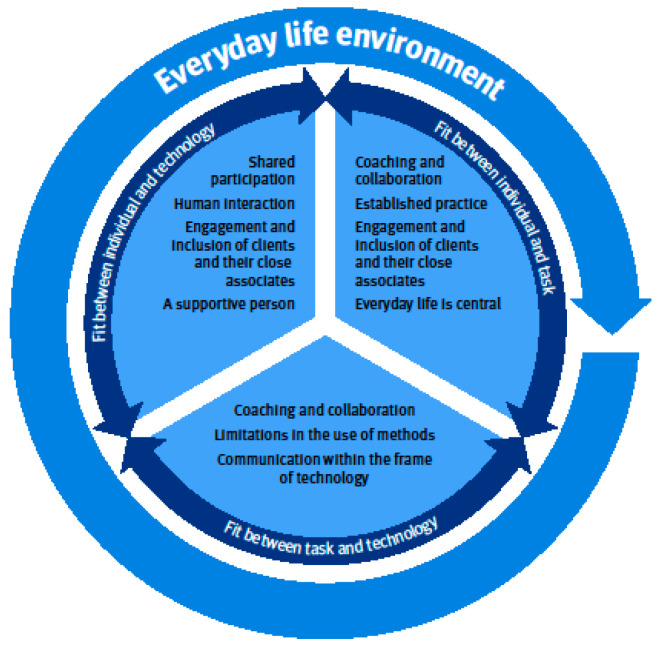
Main findings of the socio-technical dimensions affecting the optimal practice of TR.

In the first stage of the content analysis, we identified three main categories and eight generic categories. In the second stage of the analysis, we organised these categories, based on their content and by the following dimensions of the FITTE framework [20]: environment, fit between individual and task, fit between task and technology, and fit between individual and technology. When we combined the findings of data-based analysis with the FITTE framework, we obtained socio-technical understanding of the essential dimensions of TR. Main findings are illustrated in Figure 3. Main findings of the socio-technical dimensions affecting the optimal practice of TR. 

One main category Everyday-life centred and one generic category Everyday-life environment were related to the dimension environment. TR is carried out at the client’s everyday-life environment and it affects all other dimensions. Therapists needed to take the clients’ everyday environment into account when planning the use of places, therapeutic equipment, and materials. Because therapists are not face-to-face with the clients, attention has to be paid to the safety of training in everyday environments.

The fit between individual and task clarifies how therapists, clients’ and their close associates’ characteristics and tasks, or work processes with remote technology influence the therapy practice. One main category, Coaching and collaboration, was related to fit between individuals and task. The three generic categories that were related to this fit were, Established practice, Engagement and inclusion of clients and their close associates, and Everyday life is central.

The fit between task and technology illustrates how the functionality of remote technology influences tasks or work processes of the therapy practice. The fit between task and technology included one main category, Coaching and collaboration. The two generic categories related to this fit were Limitations in the use of methods and Communication within the frame of technology. TR is practiced through technology and, therefore, therapists need to adapt the methods used in therapies.

Fit between individual and technology included the main category, Shared participation. Three generic categories, Human interaction, Engagement and inclusion of clients and their close associates, and a supportive person, were related to this fit. Fit between individual and technology clarifies how therapists, clients’ and their close associates’ characteristics, and technology characteristics together influence the therapy practice. The optimal fit between an individual and technology requires shared participation and emphasises the importance of the client’s close associates’ competence to support participation in TR.

During the COVID-19 pandemic, therapists had to rapidly fit their methods to the technical possibilities of TR. Current outpatient therapies are complex and have many embedded elements that are not always easy to change. Therapy via remote contact established both practices that worked well and missing functionalities and challenges that need to be addressed. There are challenges in the TR practice, but it also brings new dimensions to the outpatient therapy.

## 4. Discussion

Based on our main findings, TR can be described as technology-mediated home-based rehabilitation. TR can integrate rehabilitation into the client’s everyday life, and it creates a new kind of shared partnership, where the focus of therapy is on coaching and collaboration. TR can provide desired rehabilitation outcomes for clients when it is based on the clients’ everyday life. Our findings, interpreted based on the FITTE framework, demonstrated environmental issues in TR by reaching into the clients’ everyday environment. Therefore, it is important to identify the characteristics of the everyday environment that may affect the ideal fit of relevant technologies. 

Previous studies have similar findings, suggesting that TR can facilitate the inclusion of the clients’ everyday surroundings in rehabilitation [15] and, consequently, enhance the integration of rehabilitation activities into the clients’ everyday lives [35]. Therefore, TR can improve the clients’ participation in their daily lives [12]. Conducting therapy in the clients’ home or other natural environment is a great advantage [11,12], and the visual remote interaction may allow the therapists to see their clients in their everyday environment [11].

Based on our findings, the focus of therapy changes towards coaching and a new type of collaboration, which requires defining the roles and responsibilities of the participants. Furthermore, previous studies suggest that remotely delivered rehabilitation can help shift the clients’ expectations away from being a passive recipient of therapies to being a more active participant in their own rehabilitation process [35,36]. However, this active role may be ambiguous to the client if the client and the therapist do not agree on it with sufficient accuracy [15].

Consistent with previous findings, not being able to conduct hands-on assessment or treatment was perceived as one of the main challenges [9,17]. Because of the hands-on nature, especially in physical, occupational, and speech-language therapies, therapists felt that outpatient rehabilitation activities are generally not easily adapted to TR. Therapists must feel competent to adapt assessments and methods and use different communication styles [8,11]. In this study, the respondents felt that TR helped to increase self-directed training and home exercises between real-time therapy appointments. However, inconsistent findings in a recent survey reported that only half of the hand therapists (55%, n = 450) asked their clients to do some form of self-directed training [7]. 

Traditionally, outpatient therapy interventions are provided on a one-on-one basis with the therapist and client. In TR, therapy becomes multilateral, and it offers opportunities to strengthen the engagement and inclusion of the clients and their close associates in the rehabilitation process. This finding is similar to a previous study suggesting that family members and other carers should be involved in TR because they can support the participation of people with physical disabilities [8]. However, some rehabilitation tasks may shift from therapists to clients and clients’ close associates with no formal health expertise or training [15], which needs to be taken into account when adopting TR. Depending on how TR sessions are practiced, TR may place greater responsibility on the client or their close associates for active engagement in the therapy intervention and, therefore, the close associates’ capacity for supporting the clients should be considered [6].

As in previous studies, these findings indicated that therapists were able to develop good therapeutic rapport with the clients [37], even though developing therapeutic rapport may take longer than with face-to-face contact [11]. These results are similar to Macdonald et al. [38] (2018), suggesting that eHealth and TR technology have been an important contributor in how client–therapist relationships have changed. Our findings are consistent with a recent study [12], in that TR should be based on the collaboration between the clients and their close associates. TR helped with the addition of relatedness between clients, healthcare professionals, and clients’ close associates [35]. Potentially, the relationships are evolving towards a more collaborative and client-centred approach. Furthermore, similarly with a previous study [9,35], integrating a close associate into TR may allow them to become more engaged than they could be in conventional rehabilitation. 

Despite the benefits of remote delivery, these findings indicate that some face-to-face delivery may still be required with clients of intensive medical rehabilitation. Previous studies have also pointed out that TR should not replace face-to-face services, especially where physical examination is required [1,9] or clients are severely impaired persons [4]. TR should complement face-to-face services [1,4,6], and it could be provided as a hybrid model with in-person and remote sessions as a means to promote the rehabilitation continuum [6]. Fitting the TR service plans to current rehabilitation processes may support and ease the professionals’ work [9,21]. To reach the desired outcome of rehabilitation in TR, the optimal use of technology is required, and that is dependent on the interaction between the key dimensions, task, technology, individual [19,20], and environment [20]. 

### 4.1. Limitations 

The current study presents findings about TR in the context of Finnish outpatient intensive medical rehabilitation at the beginning of the COVID-19 pandemic. Since this study offers insights into TR practices during this limited period, certain limitations are evident. 

A majority of the respondents were relatively inexperienced in practicing TR [3]. Professionals working in other healthcare services or who are more experienced in TR may face different challenges with TR practice. Therefore, the results may not be transferable to other rehabilitation service contexts. Another limitation was that only the perspectives of professionals were explored. To gain a more comprehensive understanding of the core dimensions of TR, other stakeholders, such as clients and their close associates, should be studied in the future as well. 

We investigated different professional groups to gain a variety of different perspectives in the adopting of TR. This can be a strength and a limitation of the study. Our aim was to form a generic description of the essential principles of TR that fit for the outpatient individual therapy practice. Due to the material containing multiple professional groups, we cannot make specific recommendations for individual professional groups based on this study.

We combined data-based and theory-based content analysis in order to gain diversified understanding of TR practice. We used quotations from different rehabilitation professionals to help confirm the connection between the findings and data. The FITT and FITTE frameworks have been used in exploring the healthcare professionals’ experiences with new technological solutions in their practice. These previous studies proposed to add environmental or contextual factors in the FITT framework [20,21], and our findings were well-interpreted with all these FITTE dimensions. 

### 4.2. Future Directions

In the future, studies should explore different client groups or other rehabilitation services to learn how we can find the optimal match between human interaction and technologically framed therapy interventions [15,16]. In addition, future studies should investigate other features of TR, for example, the long-term impact of TR [17] and on how peer support has been implemented during TR [35]. 

## 5. Conclusions

From the perspective of the FITTE framework, TR can be seen as a technology-mediated home-based rehabilitation, as it can integrate rehabilitation into the client’s everyday life and its environment. In TR, therapy becomes multilateral and it creates a new kind of shared partnership where therapy is focused on coaching and collaboration. The presented critical dimensions of TR can be useful in guiding, designing, developing, and practicing therapy interventions when shifting practice to TR in outpatient rehabilitation. Findings from this study can potentially contribute to facilitating better integration of TR into the client’s everyday life in the Finnish outpatient rehabilitation context with clients of intensive medical rehabilitation. 

## Figures and Tables

**Table 1:** Participants of the study.

Profession	N	%
Physiotherapist	249	40
Speech and language therapist	195	31
Occupational therapist	140	22
Music therapist	27	4
Neuropsychologist	18	3
Total	629	100.00

**Table 2:** Material of the study.

Open-Ended Question	Responses (n)	Pages	Words
You can share your thoughts on the impact of the COVID-19 pandemic on your work and telerehabilitation	356	47	19,959
Have there been changes in the duration and frequency of therapy visits due to telerehabilitation?	197	9	1801
Have there been changes in the goals of the therapy due to telerehabilitation?	184	9	1933
With what kind of clients has tele-rehabilitation not been a possible method of rehabilitation?	176	13	4044
With what kind of clients have you started implementing telerehabilitation?	185	14	4064
Total		92	31,801

**Table 3:** Categories identified in the data-based content analysis.

Main Category	Generic Category	Subcategory
Everyday-life centred	Everyday life is central	Part of everyday life and everyday events
		Goals based on everyday life
	Everyday life environment	Safe training in everyday environments
		Use of places, therapeutic equipment, and materials
Shared participation	A supportive person	Need for a supportive person/an assistant
		Assistant’s/supportive person’s competence
	Human interaction	Third party interaction
		Maintaining interaction
	Engagement and inclusion of clients and their close associates	Inclusion of the client’s close associates
		Commitment and responsibility
Coaching and collaboration	Established practice	Guidance and coaching methods
		Self-directed training/exercise
		Contents of therapy and the choice of methods
		Therapy practice in a technological frame
	Limitations in the use of methods	Observation, assessments, and evaluation
		Manual guidance and tactile methods
		Physical presence and shared activity
	Communication within the frame of technology	Contact is limited by technology
		The importance of speech and language skills
		Shared language

## Data Availability

Not available due to ethical reasons.
